# Autism spectrum disorder: has it lost its meaning and is it leading to misdiagnosis?

**DOI:** 10.1017/S0033291726105376

**Published:** 2026-08-04

**Authors:** Uta Frith

**Affiliations:** Institute of Cognitive Neuroscience, UCL: University College London, London, UK

**Keywords:** culture, essentialism, identity search, looping, masking, neurodiversity, subgroups

## Abstract

**Background:**

Since the 1940s, the concept of autism has undergone marked changes, and autism spectrum disorder (ASD) is a wildly heterogenous condition. At the same time, the prevalence of ASD has increased massively.

**Methods:**

This essay considers possible reasons for the changes and their consequences.

**Results:**

Among drivers for the increase in prevalence are cultural changes, such as the desire for inclusion, harm avoidance, reliance on self-report, and the acceptance of masking. These have all led to a lowering of the diagnostic threshold. Furthermore, the popularity of the concept of autism boosted numbers via self-diagnosis and a search for identity.

**Conclusions:**

Marked differences now exist between those first diagnosed in childhood and others first diagnosed in adolescence or adulthood. The time has come to examine reasons for these conceptual changes and the consequences for clinical practice and research.

## Changes in the diagnostic criteria

The definition of autism and the criteria for its diagnosis in the various editions of the Diagnostic and Statistical Manual of the American Psychiatric Association have been discussed at length (e.g. Happé & Frith, 2020). Briefly, autism first appeared as a diagnostic category in 1980 (DSM-III), based on Kanner’s (1943) classic features of autistic aloneness and insistence on sameness but added language impairments. In 1983 (DSM-IIIR), the criteria formed what became known as the triad of impairments: impairment in social interaction; impairment in communication (including language delay); and restricted, repetitive, and stereotyped patterns of behavior, retaining the core social and nonsocial features. In 1994 (DSM-IV), Asperger disorder was added as a new category. This label was applied to individuals with good language and without intellectual impairments. This presented a significant step in the widening of the criteria.

In the latest edition, DSM-V (2013/2022), the sole diagnostic category is autism spectrum disorder (ASD), which can be applied at any age and at any level of intelligence. It folds the triad of impairments into a dyad of persistent deficits in social communication and restricted, repetitive patterns of behavior, interests or activities, sticking with the core features but adding some subtle changes. Newly mentioned are hyper- or hypo-reactivity to sensory input. Furthermore, symptoms can now be met currently or by historical report, provided some impairment is present. This represents a further widening of the criteria and a lowering of the diagnostic threshold.

## Is autism real?

It is widely accepted that autism is a neurodevelopmental disorder evident from early childhood with a largely genetic basis. It is also generally agreed that while it exists on a spectrum, at its core, autism is defined by characteristic social and nonsocial impairments. But does this mean that it is a true biological entity? This is still far from agreed (Waterhouse & Mottron, 2023). Big questions are still to be asked, particularly because there are no objective biomarkers.

In the past, my colleagues and I felt confident that we could recognize autism at first impression in a thin-slice judgment (Ambady & Rosenthal, 1992). But this would not be true today in an era of record numbers of cases receiving an ASD diagnosis. Apart from the high heritability demonstrated in twin studies (e.g. Sandin et al., 2017), there is little safe ground to sustain the belief that ASD is a unifying concept that will outlast differences of opinion. This is also true for many other psychiatric conditions. It is in fact surprising how much faith we place in the ability of clinicians to diagnose such disorders.

What is meant by the autism spectrum? As conceived by Wing (1997), the autism spectrum contains a multitude of autisms that shade into each other and have a common denominator, the triad of impairments. Her notion worked on an analogy with the color spectrum of visible light; it does not run from mild to severe but is often interpreted that way.

This is not to be confused with the idea of a spectrum where there is no categorical distinction between autism and non-autism. Here, one can be a little bit autistic and definitely autistic. But this is not the same as the clinical autism spectrum, where everyone is definitely autistic, even when this is manifest in different ways.

## The uneven rise in prevalence

Why has there been a dramatic rise in ASD prevalence? One explanation is that increased awareness of autism has made it possible for previously hidden cases to come to light. It could also be that efforts at increasing mental health awareness and destigmatisation have inadvertently resulted in a rise in mental health problems (Foulkes & Andrews, 2023). However, neither explanation can account for the fact that this increase is uneven across the spectrum, across different age groups, and between males and females. We need to explain these discrepancies.

The persistence of the core diagnostic criteria over time is remarkable, but subtle changes in interpreting and applying them have lowered the bar to diagnosis. This has had a huge impact on prevalence. In the earliest population study, which assessed all children resident in Middlesex at the time (Lotter, 1966), the estimate was 4 in 10,000 (0.04%). In contrast, recent data of all schoolchildren resident in the United Kingdom (O’Nions et al., 2023) suggest a prevalence of 1 in 57 (1.76%), a 44-fold increase. For the whole population of the United Kingdom, the estimate is between 1.8 and 2% (www.gov.uk). This figure is not dissimilar to the estimated global prevalence, but, as one would expect, there are big differences between countries and regions within countries, depending on their health services.

One unforeseen consequence of a looser application of the diagnostic criteria was that individuals with intellectual disability have become a minority. A recent Swedish population study found that this group decreased from 55.8% of ASD cases in 2001 to 6.7% in 2020 (Salkic et al., 2026). This finding fits with the notion that the increase occurred in a section of the autism spectrum where intellectual disability is rarely seen.

A study carried out by Russell et al. (2022) opened a new chapter in population studies. They used the UK primary care database between 1998 and 2018, encompassing 65,665 patients who were registered with an autism or ASD diagnosis. There was a surprisingly clear finding: The increase in prevalence was specifically linked to individuals who were diagnosed after childhood, and this was true for females in particular. A US-based study (Harrop et al., 2024) confirmed these findings, indicating a steady 20-year rise in the proportion of females diagnosed with autism in adolescence or adulthood.

## A split in the spectrum

A study by Zhang et al. (2025) offered further insight, revealing large differences between individuals diagnosed in childhood and those diagnosed later. These differences emerged not only in behavior but also in their multifactorial genetic bases. The early-diagnosed profile was characterized by a high density of polygenic markers for social-communication deficits and restricted/repetitive behaviors. Conversely, the later-diagnosed profile showed a stronger genetic correlation with ADHD, major depressive disorder, and PTSD. This suggests potential diagnostic misclassification. The authors further stress that the genetic factor for later-diagnosed autism does not represent the additive genetic effects of earlier-diagnosed autism and mental-health conditions.

There are other pointers toward this split in the spectrum. Data from Fyfe et al.’s (2026) population study of nearly 3 million registered Swedish individuals born between 1985 and 2020 confirmed that females are common in the group with a late diagnosis but rare in the group with an early diagnosis. The data confirmed the well-known 3:1 sex ratio for cases diagnosed before the age of 15, while those diagnosed after age 15 approached a sex ratio of 1:1. To my eyes, this sharp turn supports the hypothesis that these later-diagnosed individuals could belong to a completely different diagnostic category, or categories.

Even though the different studies have not used identical cutoff points for early versus late diagnosis, they coincided in finding substantial group differences that were not anticipated nor compatible with the notion of catch-up, due perhaps to greater awareness of autism.

I am aware of the many attempts made to establish ASD subgroups, which have had mostly inconsistent results and disappointing clinical uptake. However, the stark differences between early- and late-diagnosed cases in population studies provide a sound basis for acknowledging two large subgroups that are so separate from each other that we can no longer see a common denominator, that is, the characteristic social and non-social features of autism. Refinements into further subgroups might be easier to accept within this initial framework.

## Drivers of changes in the autism concept

My own research in autism started in 1966, and since then I have witnessed the changes in how autism is understood, both by experts and in the popular imagination. Here I focus on possible cultural factors that might explain the rise of the late-diagnosed cases and the resulting collapse of the spectrum.

### Inclusiveness

From my own experience in the 1960s–1970s, I can say that clinicians and researchers saw only children, not adults, and all these children belonged to a subgroup of the larger group of children with intellectual disabilities. To many of us, the diagnostic criteria seemed too narrow and unfair on marginal cases. The first push toward the widening of the category, and the subsequent increase in prevalence, came with Lorna Wing’s proposal of a spectrum. This included cases with milder or somewhat atypical symptoms, as well as cases with vastly different intellectual abilities. Now we can ask: has the desire for inclusiveness eventually led to overinclusion?

### Looping

How are diagnostic categories maintained when there is no objective biomarker? There is reason to think that ASD has escaped the clinic and entered the public sphere of influence. It is no longer left solely to expert clinicians to define autism. What autistic people write in their autobiographies or portray on social media influences what appears in textbooks and in diagnostic instruments. This creates a looping cycle, as proposed by the philosopher Ian Hacking. He argued that looping occurs when a classification provides individuals with a new way of being a person, that is, a new way to experience themselves and live in society (Hacking, 2009).

### Social contagion

Social networks are an important channel for the spread of cultural memes (Neumann et al., 2026). Through a process known as social contagion, individuals may adopt behaviors they see modeled online. Social contagion is not ‘faking it’; rather, it is a survival mechanism to align with the tribe. Nevertheless, giving a medical label to the outcome of this process risks sliding into overdiagnosis. However, the surge cannot be mere contagion but may also represent a long overdue realization of unmet mental health problems. Why they are currently accommodated within the wide autism spectrum, rather than other diagnostic categories, is a matter for urgent discussion.

### Lived experience

While lived experience is incredibly valuable for understanding a patient’s perspective, an uncritical reliance on subjective experiences for the purposes of diagnosis can be treacherous. For example, it may fuel diagnostic inflation by pathologizing normal human variations. It is hard to evaluate the validity of subjective reports without a thorough comparison that takes into account direct observation and reports by others. Questions are rarely asked about the metacognitive processes demanded by self-reflection or the cultural influences shaping the language that is used to express inner experience. Intellectually impaired and nonverbal individuals are necessarily left out of these subjective narratives.

### Search for identity

With questionnaires available on the internet and an abundance of self-presentations on social media (Karpinsky et al., 2015), often aimed at adolescents, self-diagnosis has become incredibly common (Armstrong et al., 2025). We can assume that only individuals with sufficient cognitive and metacognitive abilities are likely to ponder their place in society in this way. Hence, the focus shifts away from the most impaired individuals. These dangers of self-diagnosis have been comprehensively discussed in a previous editorial in this journal (David & Deeley, 2024).

### ‘Hidden’ girls

Is the surge of females in late-diagnosed cases simply a catch-up phenomenon? If so, we should begin to see many more early-diagnosed cases of girls, but this has not happened. For decades, the sex ratio in autism has been stable between 4:1 and 3:1. Has it been a result of male-centric depictions of autism? This ignores the case of Temple Grandin, a long-held prototype of autism. A widely accepted hypothesis suggests that autism is underdiagnosed in girls and women because they show a milder form of autism or use more efficient masking (Gould, 2017). It is perhaps surprising that an alternative hypothesis has not been considered, namely that these newly emerging cases belong to alternative diagnostic categories, or a category which has yet to be given a name.

### Masking

I often wonder if the concept of masking is what opened the floodgates. Take the clause that appeared in DSM-V making allowances for symptoms that ‘may not manifest until social demands exceed limited capacities or may be masked by learned strategies’. This clause seems purpose-built for later diagnosed individuals. Yet, if masking can be used to explain the total absence of symptoms, then, in theory, this could lower the diagnostic threshold to zero.

The notion of masking differs from compensation. Compensation involves choosing alternative means to solve a task, such as learning explicit rules for otherwise intuitive social skills (Livingston, Shah, & Happé, 2019). Masking, in contrast, means concealing problems purely to blend in. How does this kind of camouflaging differ from the adaptation and impression management that everyone practices in their social interactions? The distinction seems to rely entirely on the subjective report of unease and exhaustion connected with masking. But exhaustion, fatigue, meltdown, or burnout are prevalent in other mental health conditions and can have many different causes.

### Harm avoidance

Social psychologist Nick Haslam identified a recent expansion of harm-related terminology as ‘concept creep’ (Haslam, 2016; Haslam & Tse, 2025). The expansion frames everyday challenges, which would previously not have been considered harmful, such as social anxiety or rejection, as clinical symptoms. Medical practice has also shifted toward harm avoidance, prioritizing never missing a disease, while tolerating false positives. Here lurks overdiagnosis (O’Sullivan, 2025). A diagnosis of ASD that is a false positive can easily become a self-fulfilling prophecy and is hard to undo.

Unfortunately, none of these potential drivers of change can tell us why autism has become such a popular cultural meme. Sixty years ago, the term autism was absent from public discourse. It was then so rare that only a few psychologists and psychiatrists had heard of it. Today, autism is heavily promoted in fiction and biographies, with 37 movies currently on offer (https://autism.org/autism-movies/). The internet provides endless sources of information, while social media platforms like TikTok create an autism folklore, spreading both information and misinformation (Aragon-Guevara, Castle, Sheridan, & Vivanti, 2025; Berg Egge & Gabarron, 2024).

## The tension between social and medical models of disability

The introduction of the concept of neurodiversity marked a revolutionary change for the concept of autism (Baron-Cohen, 2017). Now autism can be seen as a difference, not a disability. Hence, neurodiversity has become a powerful tool for destigmatization, celebrating the strengths of neurodivergent individuals and recasting weaknesses as challenges imposed by poor person-environment fit. Few would dispute that this framework fits the late-diagnosed group far better than the early-diagnosed one.

### The allure of the social model

According to the social model, disabilities do not reside in the individual but in a lack of societal accommodation. It is therefore wrong to ‘mourn’ a child with autism as a tragedy. Instead, the slogan promoted by advocates of neurodiversity is ‘we are not broken and we don’t need fixing’.

It’s only a small step from there to claiming a superpower. Hypersensitivity and detail focus are strengths suited to excel in certain fields, such as detecting errors in patterns or memorizing complex data by rote. This is the cause for pride. However, when autism itself is celebrated as a superpower, parents and carers of profoundly autistic individuals feel upset and bewildered.

Adopting the social model implies a shift of the burden of change from the individual to the social environment. The inspirational example is the building of ramps for wheelchair users. Yet, there is a dilemma for neurodiversity advocates. One cannot logically argue that autism is not a disorder while using a disorder-based diagnosis to claim rights and accommodations. This dilemma suggests that the movement functions more as a political and moral identity rather than a scientifically useful classification.

### The allure of the medical model

People who receive an ASD diagnosis as adolescents or adults often report overwhelmingly positive effects. They state that the diagnosis explains their lifelong struggles and liberates them from self-blame. In this way, the label itself can be therapeutic without any formal treatment. How is this possible?

For an intriguing explanation of this phenomenon in terms of cognitive processes, we can look at a study with neurotypical participants by Giffin, Wilkenfeld, and Lombrozo (2017). The authors designed vignettes involving a person who showed somewhat unusual behavior. In some, they inserted a fictitious category label referring to a mental or physical disorder to explain the behavior. These were compared to identical vignettes that did not offer a label but a vague personality tendency. Confirming earlier findings of a study by Ahn et al. (2013), laypeople responded strongly to categorical labels as reflecting a true and common cause for a disorder, valid across different individuals. This cognitive bias, known as essentialism, is present already in young children (Gelman, 2003). The essentialist bias is satisfying because it provides an explanation of behavior in general, rather than in a specific example.

Is this perhaps how we come to perceive causal explanations in named mental disorders? If so, medical labels carry a greater weight of perceived truth than personality descriptions. This would also explain why people are not satisfied with self-diagnosis and seek out a medical authority for a professional diagnosis. Only then will they feel that they have an objective causal explanation for their problems and difficulties.

## Can comorbidities rescue the spectrum?

Comorbidities, such as intellectual disability, sleep disorders, anxiety, and mood disorders, are widespread in ASD. For instance, a population-based study in Sweden (Lundström et al., 2015) found that over 50% of individuals with ASD had four or more comorbid conditions. Khachadourian et al. (2023) used the SPARK database of the Simons Foundation with over 150,000 cases and found that 74% of autistic individuals had at least one comorbidity. Comorbidities can explain why two people with the same ASD diagnosis have such vastly different life outcomes. Could differentiating cases according to these co-occurring conditions lead to individualized approaches and thus offer a way to keep the spectrum together?

At its extreme, this idea presupposes that the core features of autism can be found even if they are well hidden under layers of comorbidities. However, it is already clear that comorbidities are not equally distributed throughout the spectrum. Intellectual impairments and language delay are readily identified in early-diagnosed individuals. Conversely, anxiety and mood disorders are far more common in individuals diagnosed as adults (Jadav & Bal, 2022). Using the Danish National Register, Rødgaard, Jensen, Miskowiak, and Mottron (2021) found that psychiatric comorbid conditions were significantly more frequent in late-diagnosed cases. Furthermore, the association with psychiatric comorbidities was stronger in females. If these findings are confirmed with early- and late-diagnosed groups, holding age constant, comorbidities might actually strengthen the idea of a split in the spectrum rather than unifying it.

## Toward diagnostic precision

By continually loosening the diagnostic criteria to accommodate a vast range of social difficulties, anxieties, or sensory sensitivities, the clinical meaning of ASD has been diluted. Yet I believe that the characteristic social and nonsocial features of core autism will still be detectable in a strictly pruned spectrum.

The shift from a precise clinical into a vague, culturally amplified identity category has generated a massive demand for assessments in adolescents and adults, with an almost magnetic attraction to the ASD label. The challenge for the future is to use greater precision in the diagnostic process by giving more weight to careful objective observation, and by scrupulously probing contraindications.

We need a fresh attempt to define the specific causes of the distress and disability experienced by many of the late-diagnosed cases so we can custom-make the support they need. For example, specific therapies could address sensory issues and could prevent burnout. There are ways to reduce the symptoms of anxiety disorder and depression. There are also ways to build resilience in the face of significant social stress. None of these approaches are likely to be appropriate for early-diagnosed cases, where different types of support are needed.

Splitting up the spectrum would ultimately prevent misdiagnosis. New labels may appear, and different specialists are likely to be needed, at least for the two large subgroups that are now emerging. Such a reorientation would signpost a new route toward the correct support for many of the individuals who are on the expanding waiting list. At the same time, it would counteract the tragic neglect of those with the most severe impairments.
